# From comfort zone to front-line care: perspectives and reflections of community pharmacists entering home-based palliative care

**DOI:** 10.1186/s12904-023-01332-z

**Published:** 2024-01-03

**Authors:** Chien-Yi Wu, Yu-Hsuan Wu, Yi-Hui Chang, Min-Shiow Tsay, Hung-Cheng Chen, Yu-Ling Kuo, Hui-Ya Hsieh

**Affiliations:** 1 Department of Family Medicine, School of Medicine, College of Medicine, Kaohsiung Medical University, Kaohsiung City, Taiwan; 2 Department of Family Medicine, Kaohsiung Medical University Hospital, Kaohsiung Medical University, Kaohsiung City, Taiwan; 3 Doctoral Degree Program of Public Health, College of Health Sciences, Kaohsiung Medical University, Kaohsiung City, Taiwan; 4 Department of Nursing, Kaohsiung Medical University Hospital, Kaohsiung Medical University, Kaohsiung City, Taiwan; 5 Department of Obstetrics and Gynecology, Kaohsiung Medical University Hospital, Kaohsiung Medical University, Kaohsiung City, Taiwan; 6 Department of Specialist Nurse and Surgical Nurse Practitioner Office, Kaohsiung Medical University Hospital, Kaohsiung Medical University, No.100, Shih-Chuan 1st Road, Sanmin Dist, Kaohsiung City, 80708 Taiwan

**Keywords:** Community pharmacist, Home care, Palliative care, Opioid, Qualitative research

## Abstract

**Background:**

Palliative care requires a multidisciplinary team to assist patients and their families to obtain good quality care at the end of life. Typically, community pharmacists have fewer opportunities to provide services for patients with palliative care needs than hospital pharmacists. Moreover, home-based palliative care (HBPC) by pharmacists remains low and there is a lack of research regarding HBPC provided by pharmacists. Therefore, this study sought to understand the views and reflections of community pharmacists in the clinical frontline providing palliative home services.

**Methods:**

Purposive sampling was used to recruit six community pharmacists for one-on-one, in-depth, semi-structured interviews and the data were analysed using thematic analysis.

**Results:**

Five major themes emerged: [1] Engagement, [2] Challenge, [3] Mission, [4] Career metamorphosis, and [5] Outlook. The pharmacists described how they engaged in HBPC and faced the challenges. They regarded opioid management as a burden. Moreover, some mentioned that reimbursement for palliative home care is low or non-profitable. They suggested building a platform to exchange advice and legislation adjustments so that they could pass on their experiences to less experienced pharmacists in HBPC.

**Conclusions:**

The involvement of pharmacists is crucial to provide better palliative care. Although the present study was small and might not fully represent the whole situation, the findings could still inform future education, training, and policy planning to promote pharmacists’ participation in palliative care to generalise community palliative care.

## Introduction

Palliative care could benefit patients with life-threatening illnesses and their families to achieve a good quality of life [1]. Although physicians and nurses primarily provide the most palliative care, pharmacists play the leading role in palliative care needs, polydrug dispensing, and health education [1–4].

Traditionally, a pharmacist’s primary role involves receiving and dispensing prescriptions, assessing the patient’s physical condition, organ function, medication status, and potential drug interactions, confirming the identity of both the patient and family members, and offering medication education during dispensation. However, when tending to patients requiring palliative care, pharmacists must not only adhere to conventional care methods but also fully utilise their practical value due to the heightened complexity and unpredictability [3, 5–7]. Moreover, dispensing and providing health education on opioids pose additional challenges for pharmacists [8–10].

Generally, community pharmacists primarily dispense simpler medications, unlike hospital pharmacists who frequently handle more complex pharmaceutical services, resulting in fewer opportunities for community pharmacists to overcome challenges related to palliative care assistance [11]. However, there is an increasing demand for palliative care today [12, 13] and palliative home care is vital to give patients in the community access to palliative care and reduce medical expenses [14–17]. The effective implementation of palliative home care in the community depends not only on the participation of physicians and nurses but also on the input of community pharmacists [8, 18–21].

In Taiwan, home palliative care was initially only provided by the hospice team attached to the hospital but category B palliative home care, mainly provided by community physicians and nurses, was introduced in 2014 to expand the scope of hospice and palliative care [22]. Since then, community pharmacists have been duty-bound to participate in ensuring the smooth delivery of palliative care in the community. Home pharmaceutical care is important and effective but pharmacists’ engagement is still based on policy compliance, hospital promotion, guidance, and encouragement [23–27].

Nevertheless, despite well-intentioned policy design, palliative services provided by hospital hospice teams still account for the majority of hospice services in Taiwan [28]. Few studies have explored why community pharmacists are willing to invest in home palliative care services and the challenges of opioid dispensing and home pharmacy services for palliative care patients. Understanding these might significantly encourage community pharmacists to invest in home palliative care.

Recently, many community healthcare staff have been willing to devote themselves to community hospice services [28]. It is an essential and valuable experience for community pharmacists to step out of their comfort zone of simply dispensing in pharmacies and devote themselves to the delivery of medicines and participation in palliative home care. Due to the small number of community pharmacists involved in home-based palliative care (HBPC) and the limited research on their experiences, this study explored the issue through qualitative in-depth interviews to understand the process and experiences of community pharmacists’ professional involvement in HBPC.

## Methods

### Design

This qualitative study was conducted through in-depth interviews and the reporting of this study complies with the consolidated criteria for reporting qualitative research (COREQ) recommendations [29].

### Participants

Due to the low participation rate of pharmacists in HBPC in Taiwan, a purposive sampling method was adopted to recruit pharmacists in southern Taiwan who had opioids in pharmacies and were engaged in community palliative care. Inclusion criteria for participants were (1) a professionally licensed pharmacist who had participated in community HBPC; and (2) had been in HBPC for at least six months. Potential participants were contacted by phone or email and explained the purpose and context of the study. An interview appointment was then scheduled if the pharmacist agreed to participate.

### Data collection

After the participant provided written informed consent, a face-to-face, semi-structured, in-depth interview was conducted in a quiet place such as a café or meeting room of the participant’s choice from 15 October 2021 to 15 November 2021. Six community pharmacists were invited to be interviewed and no participants refused or dropped out of the study. All participants collaborate with the palliative team from the southern territory hospital where C.Y.W. works. Each interview generally lasts 40–65 min. Participant characteristics and interview length are shown in Table 1. A semi-structured interview guide was used [28] and the participants were also asked about the barriers to dispensing and management of opioids in the community. The interviews were audio-recorded, and C.Y.W. and Y.H.W. took field notes to reflect on any issues raised during the interviews.Follow-up questions were asked based on the participants’ responses. Since the six participants were all those involved in opioid regulation and community palliative care services in the city, no further participants were sought. Interview records were then transcribed verbatim. Transcripts were not returned to participants for comments or correction, and no repeat interviews were conducted.

**Table 1:** Participant characteristics and interview length

Participants	Education	Working year	home care year	palliative care year	interview length (min)
A	Master’s	37	4	4	64
B	Bachelor’s	26	3	3	65
C	Master’s	30	6	5	40
D	Bachelor’s	20	6	5	60
E	Master’s	20	4	2	60
F	Master’s	6			

### Analysis

Manual techniques and analysis software MAXQDA (version 12.0.3) were used to organise text fragments and coding. The data were analysed using thematic analysis which involved transcribing the data, repeated reading, noting meaningful concepts into codes, grouping codes into themes, and reviewing and renaming themes. For example, the statement, “Our first palliative-related work was to develop an integrated home-based care plan, and as health authorities established a pharmacist delivery reimbursement scheme, we began medication home delivery, medication integration, and counselling services….” was coded under the subtheme “Fresh turning point.” This subtheme was later merged with another related subtheme, “Embedded in the community,” into the theme “Engagement.” Discussions with the research team, including C.Y.W., Y.H.W., and H.Y.H., were conducted during coding and interpretation to avoid bias.

## Results

Five themes emerged from the data analysis including (1) engagement, (2) challenge, (3) mission, (4) career metamorphosis, and (5) outlook (Table 2).

**Table 2:** Study themes and subthemes

Themes	Subthemes	Subthemes	Subthemes	Subthemes	Subthemes
**Engagement**	Fresh turning point	Embedded in the community			
**Challenge**	Barriers to Opioid Management	Problems when performing home-based palliative service	Chores for non-pharmaceutical professional services		
**Mission**	Multiple and inappropriate medication use	Medication Hoarding	Monitor Compliance	Medication Costs	
**Career Metamorphosis**	Being shocked	Proficiency	Mind changing	Becoming a family Pharmacist	Showing self-valued
**Outlook**	Building a connection platform	Legislation adjustment	Pass on experience and the flame of passion		

### Engagement

Nearly every participant mentioned their willingness to serve the community as the main reason for their participation in HBPC, in addition to the overall trend of Taiwan’s ageing society and the policy promoted by government agencies.

#### Fresh turning point

Although some respondents said the reason for joining community HBPC was to cooperate with the policy promotion of government agencies, some respondents noted that it was mainly because they felt the needs of those around them:

*“Instead of waiting, I opted for home-based palliative care without the support of the Health Department and Medicare regulations, which state that pharmacists who practice home palliative care are reimbursed.” (C)*.

*“I once watched a movie about home-based care, which had a very, very big impact on me. I have watched it four times in a row, and it made me think, what are a person’s dignity and expectations at the end of (life)? When encountering some patients, I often hear their family members saying the patient may want to go home; however, they don’t know what to do when they return home?” (A)*.

*“Our first palliative-related work was to develop an integrated home-based care plan, and as health authorities established a pharmacist delivery reimbursement scheme, we began medication home delivery, medication integration, and counselling services….” (E)*.

*“Initially, the Ministry of Health’s long-term care unit advocated home-based palliative care. However, managing opioids has become an issue for the community, so we are entrusted with it.”(B)*.

#### Embedded in the community

Interviewees said they would partner with a hospital near their pharmacy to provide door-to-door medication delivery service. In addition, vulnerable patients often require more home delivery services due to the lack of help from their families:

*“Because my pharmacy is near City Union Hospital. When the hospital started promoting home care, it wasn’t convenient for some patients to visit the hospital for their prescribed medication. So they were seeking a pharmacy in this area that could deliver medicine to their home, and that’s why I started such a service.”(B)*.

*“There are a lot of vulnerable populations around this area who may not have a carer. People like this have nobody to assist them in accessing medication from the hospital. So we invested in the medication home delivery service. However, not all pharmacists are willing to do such service for free.” (B)*.

### Challenge

For HBPC, most respondents indicated that the difficulties and challenges arose from the administrative work rather than from the professional aspect of pharmacists. These administrative tasks, such as medication management, staffing, and medication costs, put pressure on pharmacists.

#### Barriers to opioid management

Community pharmacists’ most significant challenge in HBPC was applying and managing opioids which was multifaceted.

*Cumbersome administrative procedures* The administrative procedures for community pharmacists to apply for opioids and their increments are cumbersome and lengthy. Increasing the preparation dosage is often necessary due to the patient’s medication needs. However, due to the cumbersome application process and the lack of immediate access to the medication, this can lead to unmet pain control needs of patients putting pressure on pharmacists.

*“The administrative management of morphine in the incremental application process is very trivial, time-consuming, and sometimes too late to provide medications to patients. Additionally, the details of each prescription must be scanned and sent to the Regulated Drugs Administration. Things like this put much weight on us, so I don’t think many pharmacies can do palliative service because of the complicated process.”(B)*.

*“The most common problem is the preparation of morphine medications. Most of the issues encountered in home-based palliative care inspections are related to this schedule, which is troublesome. One is the official documents process, which is necessary for safety reasons. However, I can place the next order only after I have placed an order and after it arrives. Meanwhile, the doctor might increase the dosage while waiting due to the patient’s condition. If it is necessary to increase, I have to let them wait. But the thing is, pain is difficult to control.” (E)*.

*When our medicine is insufficient, I need to apply for more. It takes at least a week to go through the process. But the thing is, our patient needs the medication urgently……. That’s the time we are worrying.” (C)*.

*“I think everyone might be terrified to manage opioids because it’s troublesome, um, it’s the process I just said, and that if there is no medication now, it will be very troublesome. We won’t know what to do. ”(F)*.

Pharmacists also mentioned that they need more staff to assist and be involved in medication delivery services because of the cumbersome management of opioids.

“*You’ve got to have enough staffing because opioids management is a big issue.”(B)*.

*" It(opioids) needs to be registered, and if there’s one missing, you look for it all day……keep thinking like where it went? “If you accidentally drop a vial of morphine, the reporting process could be so complicated… and you must always be tense. Furthermore, you must count the amount of the fentanyl patch every night to ensure the count remains correct.”(D)*.

*Non-transferable* The opioid drug amount is controlled annually and needs to be appropriately allocated by the drug supervision and management department. However, opioids cannot be transferred between hospitals and community pharmacies due to legal restrictions. Pharmacists mentioned that in the early days of palliative care services, many opioids expired because they did not have enough patients requiring them. It is evident that the flexibility of rational drug use is a big problem!

*“In the first year, I prepared three kinds of opioids, but we didn’t use them for a whole year, then went to the health centre to destroy them…it was a waste. If we have many community pharmacies now, but not enough palliative care patients, morphine would expire and become wasted because they cannot be transferred to other units” (B)*.

*“The Department of Drug Administration has regulations on the production of opioids. For example, how many people have cancer by population, and what is the proportion? Then they will take an inventory of these drugs and decide what to produce this year. Then they will distribute the doses evenly. Not just in specific settings. There were no cases at first when we started palliative care services. As a result, the opioid drugs were thrown away in the first year because there were no cases. The opioid transfer system has become a big issue for us.” (C)*.

#### Problems when providing home-based palliative service

Compared with typical pharmacist dispensing services, home-based palliative pharmacists have relatively more requirements and restrictions, affecting the investment threshold of home-based palliative pharmacists that may dampen their enthusiasm to participate.

*Distance limitation* According to health insurance regulations, the delivery distance is regulated but because there are currently only a limited number of community pharmacists willing to invest, everyone still tries their best to meet the needs of patients with the permission of government agencies, which is also an intangible cost for pharmacists.

*“Palliative patients in urban areas will not be short of medicines because they can go back to the hospital to get them. But those who are a little far away from the urban area, or those who are vulnerable with no one who can assist in getting medicines, are the ones who need the service. However, some medicines have been delivered farther than ten kilometres away from the home medical integration range of the National Health Insurance Department. So I also specifically asked the health insurance department, and people there said it’s okay because very few people are doing it now, so we don’t exceed ten kilometres in principle. ”(B)*.

*“There are some uncles and aunts who have limited mobility. It may be inconvenient to come to get medicine. But to be honest, whom we deliver medication to their homes, most of them live near us.” (C)*.

*Identity restriction* For home-based palliative pharmacy services, the requirements and regulations may also lead to low participation of pharmacists.

*“ Only three types of patients can receive medication delivery services and evaluations. One is who uses opioids; the other is who has a specific form of the medicine, such as an inhalant or injection. The last one is that physicians considered the patient needed to be referred to a pharmacist. The terms of the referral are strict.” (B)*.

*“By delivering the medicine and then assessing the use of the medicine, you can report a professional fee. But there are restrictions on identity. First, you must complete the home pharmacist care course organized by the National Federation of Pharmacist Associations and pass the exam and internship. And the second is that your pharmacy must have more than two pharmacists. So this restricts the joining of many pharmacists. So it is difficult for pharmacists to implement home care service.” (B)*.

*“In terms of application fees, the regulations stipulate that the pharmacy must have two pharmacists, so one can go out for service? Both of them need to meet the qualification threshold” (C)*.

*“We just want to say that we can (serve) the community as much as possible because I know that very few people in the community pharmacy are doing this. The criteria for hospice service are relatively strict, and it is inconvenient to manage opioids. What’s more, the realistic consideration is your financial status. The drug price is high, and it has to be delivered timely. The official documents and ordering process will differ from the general medication preparation.”(E)*.

*Service amount limitation* Pharmacists can only provide limited home services each time, and the source of cases often depends on referrals from home care teams which will affect pharmacists’ income and willingness to invest.

*“We pharmacists can not actively find our cases but rely on the team referral, which means all we do is wait and wait and wait…”(B)*.

*“A community pharmacist can exert a relatively large influence because of the local value. It would help if you had more staff to do it. You can’t close your pharmacy for the home delivery service since people are still coming for medication. On the one hand, you want to promote home medical care, and on the other hand, you want to support the public interest.” (B)*.

*“You need manpower; otherwise, when you go out, you can usually only deliver one or two in a morning. There are not many cases, ah. If I rely on this, I will “starve to death,” and I don’t even dare to count the profit I made.”(C)*.

*Unprofitable* Dispensing opioid drugs is unprofitable for pharmacists, and there is no additional payment for home medication delivery services. Furthermore, a particular qualification is required for pharmacists involved in home medication delivery service, thus pharmacists consider participating in HBPC as a losing business.

*“Opioids are essential for home-based palliative care, but on the one hand, the policy on controlled medications here is stringent. Second, to be realistic, there is almost no profit doing controlled medication delivery.” (A)*.

*“In fact, there are no profits in providing and delivering home care medication. It’s such a losing business.” (B)*.

*“If you tell other pharmacists what pharmacy services you provide, you’ll be scolded, saying it’s all because you do such service for free so that people think pharmacists don’t lack money. Reimbursement rules are strict for home health pharmaceutical matters. The qualifications for pharmacists to join the palliative care team are also challenging” (B).*

*“It is unprofitable to do this (distribution of morphine-like controlled drugs and home medication delivery); we can only make up the profit with our pharmacist service fees.” (E).*

#### Chores for non-pharmaceutical professional services

Many interviewees also mentioned that in addition to their speciality, they often find that the public needs assistance with other problems which will also affect the quality of patient care. Although they want to assist them with referrals to other services, they still feel helpless whenever patients or families refuse to accept their help.

*“The government also has a platform. It has other occupations that can make referrals, but the premise is that the family members agree. For example, if the patient needs nutrient service, I would refer them to a nutritionist. But the problem is, the family disagrees with it …Sometimes there is only a foreign caregiver left, and then the language barrier and the patients are grumpy and emotionally unstable… I don’t know whom to teach” (E)*.

*“Maybe sometimes you will find that the patient has no major physical problems but needs social help. Sometimes we want to seek help from social workers, but we don’t know whom to ask for help.” (F)*.

*“During this service period, we felt the patients’ problems are not only about medications, but some nutrition-related dietary advice from a nutritionist, rehabilitation, and even psychological resources. They do not have these resources. It should be said that generally speaking, after seeing the doctor in the hospital, they come to the community pharmacy to get medicine…this is not enough.” (C)*.

### Mission

Many participants mentioned that they only discovered the problems with many patients’ prescriptions after the door-to-door service, which has implications for public health and national health policies and systems. However, community pharmacists also found that this task must be undertaken and demonstrated by their profession.

#### Multiple and inappropriate medication use

Physicians and community pharmacists may also find potential multiple and improper medication use in the community during their daily services.

*“I once encountered a special case. There was an older lady who suddenly lost 5 or 6 kilograms. She took no special medicine but only seven to eight kinds of supplements. Then I told her to bring them to me to check up. You know what? I was shocked after I saw what she had been taking. All of them were for joint-supplementing glucosamine. She bought them online, her neighbour gave her…her daughter bought them for her, and his son repurchased them… She ate all of it at once every day… she’s been taking six times doses! That was insane!” (A)*.

*“Only when I visited their house did I find that there were a bunch of medicines. The ones that came from here(hospice home care) all came from there(other hospitals and clinics)” (D)*.

#### Medication hoarding

Many interviewees thought the patients would take the medicines according to the doctor’s advice. However, when they were engaged in home-based pharmacy services, they discovered that patients typically hoarded medicines.

*“Visiting the patient’s home is recommended because sometimes he does not want to show you the spare medicine. You won’t know anything else unless they take the spare medication to the pharmacy for you to throw away. You might still think they’ve taken all their meds.” (D)*.

*“When you see the situation at home directly, sometimes what the patient says differs from what you see… He knows how to take these medicines. He said he is also taking them, but when you arrive, you will see a sack full of medicines over there…” (F) *.

*“I once met a home care patient. His medicines were stored for nearly two or three years…, he didn’t take them, and he kept asking doctors to see him. He thought that the medicine given to him would make him ill. He won’t take it, but he always wants the doctor to come and see him.” (A)*.

#### Monitor compliance

The interviewees mentioned that as a front-line community and home-based pharmacist, it is easier to perceive the significant gap between the patient’s medication compliance and the actual situation. Furthermore, some pharmacists can understand the factors of poor medication adherence and resonate with the patient’s sentiment.

*“A bunch of medicine that he didn’t take because he didn’t feel like taking the meds….” (D)*.

*“The degree of cooperation with medication is different from expected. And then there are more shocks, which means that you will have a lot more empathy to understand their situation such as why he doesn’t take it, why his treatment may lead to failure …There are a lot more emotional considerations” (E)*.

*“Many uncles and aunts have a lot of spare medicines at home. This means that most patients are not taking their medication as prescribed…And why is that? There are various reasons, such as inconvenience, timing issues, or side effects. However, they may not have told their doctor and taken the medication in their own way. Of course, this will affect the treatment.” (C)*.

*“I think the community pharmacy is more convenient in easy access to patient consultation, and it should be utilized well. The part of home care, of course, is an even better resource… I once met a person who received home care treatment and told me he didn’t take the doctor’s prescribed medication. I was surprised that he didn’t use it. He said he went to listen to the “god’s medicine” and the folk prescription the neighbour next door reported to him. I said to him, “You might be wrong. You should be obedient to the doctor.” Later he accepted what I said… Community pharmacists should walk in the community and give people better consultation channels.” (A)*.

#### Medication costs

Respondents mentioned that if community pharmacies can invest and maintain the same quality of medicines as those obtained in hospitals, it will make it easier for patients and their families to receive home care, resulting in significant savings in overall medical costs.

*“The investment of the community pharmacies…is to allow patients and their families obtain home medical services more efficiently, and try to make the medical treatment process equivalent to the hospital… This can meet the medical effects patients expect, make them feel comfortable, and reduce medical expenses in Taiwan. Pharmacists can be another medical profession’s gatekeeper to prevent people from taking their medications inappropriately ”(A)*.

### Career metamorphosis

Compared with the service content of simply dispensing in the past, pharmacists can only feel the actual clinical situation and improve the profession by stepping out of the pharmacy and entering the home care service. Also, pharmacists could show their professionalism by providing palliative home care.

#### Being shocked

The interviewees mentioned that only by walking into the patient’s home and seeing the changes in the patient’s condition can one truly understand what is genuinely “clinical.“

*“Generally, the patient we deliver door-to-door medication lives near our pharmacy. The patient will know who you are and will not particularly regard you as a professional occupation because they are used to you in their community. As for the hospice part, sometimes the patient’s condition will change suddenly, which I have never seen before…since we did not have to care for any patients before… and I have never seen the manifestation of dying progress in the front line. We participated in many meetings, checking whether the patient was taking high-risk drugs and having liver and kidney functional changes… I later felt that what I did before was not called ‘clinical’…” (B)*.

*“There was another time…the patient was breathing fast…… just kept twisting himself and moaning all the time, ahh… that was a great visual and psychological impact on me.” (B)*.

#### Proficiency

Respondents believe that although home palliative care procedures and medication preparation are relatively complicated, the whole process will become more proficient if the team constantly communicates and implements. Due to the accumulation of experience, respondents can also perceive clinical changes in patients by adjusting medication dosage or changing prescriptions and responding accordingly.

*“Later, we now have a certain sensitivity, such as adjusting the drug and increasing the dosage of morphine. We can probably sensor the progress of the patient’s condition through the change of the drug prescription, and then we will adjust the direction of health education…” (B)*.

*“I think administering opioids can be cumbersome and even overwhelming when the needed dose rises rapidly. I believe this can be resolved with administrative intervention. let the government loosen the restrictions. Of course, as a community pharmacist, when you are familiar with the process of everything, you will find that it is not difficult. ”(A)*.

##### Mind changing

The interviewees mentioned that although it is very tiring, they can drive themself to continue providing home care services and find professional value through mind changes. Furthermore, they could show their professionalism and value, become the team’s backup, and make patients and families feel at ease by providing HBPC.

*“The distance to deliver the medicine is too far, yeah…too far… Sometimes I would think, ‘Why must we provide this service? This service is unprofitable…’But, on the other hand, what will doctors and nurses do without us if we do not provide such a service? What if the patient can’t get outdoors for medicine and is dying of pain?” (B)*.

*“Each additional controlled-drug application requires much administrative work, making me want to quit doing it. But whenever I think again, ‘What if I do not do it? What about the patients’ pain?’ Ultimately, I just kept doing it (requiring more controlled-drug)every time.”(B)*.

*“Later, we also discovered some models. For example, through medication delivery, we went to many places in Kaohsiung that we hadn’t been to yet… Oh, it turned out that the urban area is stunning. It’s good to get to know Kaohsiung again through delivery service.” “So I am like… let’s keep doing it unless they don’t need it, or there are too many people doing it, then maybe there will be a hint to you, that is, you can stop this matter. If this never happens, keep going, which is my value.” (B)*.

*“Doing pharmacist service is to warm people’s hearts, and also you have to think about it, that is, what kind of care you want when you are old, then you should provide such care of others first.” (E)*.

#### Becoming a family pharmacist

Community pharmacists not only pursue their profession but also improve their ability to provide whole-person care and transfer people to the care resources they need in a timely manner.

*“Community home care is still mainly based on the diagnosis of doctors and the care of nursing staff. In the process of giving medication, we still have to track his follow-up curative effect and the use of specific medicines… we can intervene to help patients understand the methods and timing of taking medication to achieve the therapeutic effect.” (A)*.

*“When people don’t know what resources they’re looking for, they come to us regarding resources like home care or long-term care.” (B)*.

*“Honestly, I see the value of being a home-based palliative care pharmacist. After you try it, you come across all kinds of illnesses and families…and also, you see relationships or family relationships of the terminally ill… it’s like reading their life reports. ”(B)*.

*“If we do it like a typical community pharmacy, there are already many similar ones in Kaohsiung City… then what is the difference or competitiveness between us and others? We have to do whole-person care, which includes different aspects… so we can make people consider us as their family pharmacists; no matter their problem, as long as it’s health-related, they can come to us for consultation. Yeah, this is our ultimate goal”(C)*.

*“Sometimes we find their (patients’) needs including long-term resources through conversation, then we will proactively give them related information. (Home Care Center)” (C)*.

*“I just had a case and found out that he needed home care services…he came to get his medicine, and then we found him living alone, far from here, which was inconvenient. So we introduced him to home care services.” (F)*.

*“Because we work with some nursing homes and long-term care centres, we provide them pharmacist services; the community pharmacy will be more convenient because our hours are flexible, then the pharmacists are all there, we can negotiate and communicate easily…” (E)*.

##### Showing self-valued

An effective medication supply chain depends on the pharmacist’s willingness to invest and prepare enough drugs to provide the physician with a prescription, a vital support for the palliative team. Pharmacists offer clinical services, including patient education, medication delivery, and proper medication management. Respondents feel they could leverage the pharmacist’s strengths in interdisciplinary palliative team care to embody their values and provide peace of mind for patients and families.

*“Not every pharmacy carries controlled drugs…and that bothers physicians on the palliative care team. We then work together to ensure adequate medication is available throughout the palliative care period and to provide patients with medication as quickly as possible”(C). “That is to say, if we set up a system to do medication passing, it would allow patients to get medicines, and doctors can prescribe medicines without worries. Our pharmacy could become a strong backup” (C)*.

*“Generally, what medication they need, we will provide and deliver to the patients no matter how late it is…I mean, it’s just to make them feel secure. The physicians are very kind to us since they need our service… I think the collaboration is good” (D)*.

*“As patients use narcotics, there will indeed be a lot of gastrointestinal discomforts and dizziness… I think with us (pharmacist) involved, patient and family will feel more secure.” (B)*.

*“Through home-based palliative care, you can report the issue whenever you see any problems and get feedback immediately. For example, when I was delivering the medicine, I found that the patient had too much medicine remaining… so I contacted the team immediately, and they’ll make sure not to prescribe a lot next month they visit…I mean, I am also contributing to the country because I am reducing medical costs. And then I realised that pharmacists are important in the team.” (B)*.

## Outlook

The health authorities should adjust and establish corresponding platforms and related policy systems to increase the investment and participation of community pharmacists in HBPC. In addition, arranging experience exchange, education, and training of the pharmacist association will improve community pharmacists’ service capacity.

### Building a connection platform

It would be convenient to share various professional caring information with team members and respond in a timely manner through an information transmission and communication platform.

*“We usually join the team chatroom on the app LINE, including me, as well as the doctors and nurses in the home care centre. I will reply to the messages if they have questions about medications. That gives me the idea of having a platform that allows the primary medical settings such as clinics, nursing homes, community pharmacies to connect….”*

#### Legislation adjustment

Based on their practical experience, the respondents indicated that it is necessary to adjust relevant policies and systems to facilitate community pharmacy pharmacists to invest in HBPC. Furthermore, they emphasised the need for editing the morphine-like controlled drug transfer rules to address the issues and increase the willingness of community pharmacists to participate in HBPC.

*“I hope the regulations can be more flexible such as a faster application process and the transfer system for controlled drugs. For example, the controlled drug I ordered has come, but the patient has passed…then I will be worried about the residue. But meanwhile, another pharmacist might lack that medication…If the transfer regulation is flexible, I can pass the mediation to him. and Problem Solved!” (C)*.

*“I think the transfer system is a good solution because it will lower the drug costs and ease your burden. And the drug would be more punctual! If there were many pharmacies today willing to participate in this project or communicate with each other, I believe that we pharmacists would not have so much pressure to work emotionally and financially.” (E)*.

*“The logic is to make it(home-based palliative care) more common…It is like painting; you can’t only focus on one spot…you’ve got to even the resources! There is no way for pharmacists from the big medical centres to deliver medication, but yep, that’s the current situation now. Most resources are in the big ones(medical centres) because the system hasn’t changed…” (D)*.

#### Pass on experience and the flame of passion

Junior pharmacists could gain valuable information and advice regarding investing in HBPC from the experiences of senior pharmacists:

*“Some are like we are initially opening a pharmacy or dealing with administrators. How do you apply for the whole process? Some pharmacies may be unable to find the information first, and then there are many steps, so Pharmacist C is only experienced. He does a very simple, what looks like a PDF file, a flowchart, and then we run through the process, and after it’s run, we start ourselves, so we’re talking about the information part, yes, at least you’ve been asked to know how to do this a little” (E)*.

*“I believe many pharmacists are willing to step into it(palliative care) but just not knowing how to start. We also suggest that the related associations can hold lessons to share information and to pass on their experiences. The stories would encourage newcomers to invest in home-based palliative care.”(A)*.

*“To take care of individual cases, we need to learn how to deal with them and how to talk to them. Our behaviour is critical. There should be no neglect. I believe that the inheritance of this experience is significant.”(A)*.

Respondents also mentioned that they need to go beyond the framework of traditional pharmacist services and return to their original aspirations, be willing to contribute, and have enthusiasm for participation. By implementing home-based palliative services, they can be more familiar with these services, further enhancing the value of their role as pharmacists. What motivates them to serve is the feedback from patients and their families, and that spirit will be passed on to other junior pharmacists.

“You will feel that participating in the team, you can learn a lot from each other, and there are many aspects that we have not thought about before…” (C)

“*If you have passion, keep doing it. Working in the medical field requires a little more enthusiasm; don’t forget the original intention of being in the field. Then as a role (pharmacist), do your best. Don’t limit yourself. Try not to say no and take every opportunity to serve. After all, I think patient and family feedback motivates you to keep doing this.” (E).*

*“To promote these ideas(home-based palliative care), it may be more useful to talk to young pharmacists when they are just entering the field and are still enthusiastic.” (B)*.

*“It’s like an important seed in the pharmacy field that can gradually influence more and more junior pharmacists to join home care and even palliative care. We have a care motto: do what we’re supposed to do. That saying will forever be etched in me in my mind.” So in the home care field, I believe I will keep my feet on the ground and never back down in my lifetime. (A)*.

## Discussion

Our research shows that community pharmacists can go beyond traditional pharmacy dispensing and engage in HBPC. Five themes emerged from the interviews with pharmacists involved in HBPC including (1) engagement, (2) challenge, (3) mission, (4) career metamorphosis, and (5) outlook.

The first theme is engagement, encompassing the occasions and contexts in which community pharmacists participate in HBPC. Although government promotion is a key factor for their involvement in HBPC, the most significant motivator is the desire to contribute to the community and deliver specialised pharmacy services. Only after participating did they experience various scenarios of home palliative care and face numerous challenges and difficulties in preparing morphine-like medications. However, after going through these home service processes, they felt a sense of satisfaction in being a home-based pharmacist and found that they demonstrated their unique value and were fulfilled being part of the community health care team. Finally, pharmacists mentioned the prospects of HBPC.

Regarding the origin of pharmacists’ participation in HBPC, some respondents were willing to invest themselves. Coupled with the country’s adjustment to the HBPC policy, the payment of pharmacists has begun to be included in the scope of home-based medical care, which makes it reasonable for pharmacists to participate in home-based care. In addition, affected by factors such as regions and patients, pharmacists are willing to deliver medications to their homes and provide HBPC, resulting in community pharmacists participating in HBPC.

Past research has not explicitly explored why community pharmacists engage in HBPC but community pharmacists’ involvement in community HBPC is necessary and long-standing in some countries, showing that community pharmacists must play a vital role in investing in and participating in local palliative care [8, 10, 18, 20, 21, 30]. Therefore, national health authorities should actively encourage and support community pharmacists to invest in HBPC to benefit the implementation and popularisation of community palliative care.

In engaging in community home palliative services, many interviewees mentioned that the difficulty in implementation is due to the various challenges of administrative issues, including medication management, staffing, and medication costs, which increase the burden on pharmacists. In particular, community pharmacists have difficulty with the process of application, purchase, and disposal of opioids, thus, there is an apparent resistance for community pharmacists to participate in HBPC. Other studies have also mentioned this phenomenon, indicating that opioid issues are challenging to obtain. Cumbersome regulations on distributing and selling opioids will hinder the promotion and development of community palliative [9, 30, 31], therefore health authorities should consider how to be more flexible in the management of controlled medications in community pharmacies since the use of opioids is critical to the quality of palliative care. In addition, the delivery of HBPC by community pharmacists puts tremendous pressure on community pharmacies with limited staffing. Health authorities should also consider how they can help improve the problem. Similar predicaments have been mentioned in only a few foreign studies [31], but this is also a critical point that cannot be ignored for HBPC in local communities.

When community pharmacists are genuinely engaged in home-based palliative services, they can proactively discover patient medication problems on the front line and identify factors that significantly impact patient care. These would not occur if the pharmacists did not conduct face-to-face visits. Previous studies mentioned that pharmacists’ consulting and engagement with patient care can effectively improve medication adherence and the overall quality of care [32–34], pointing out the importance of pharmacists in the palliative care team.

A pharmacist’s profession goes beyond dispensing and providing basic medication instruction in pharmacies. Entering the field of HBPC could implement the spirit of inter-professional cooperation between doctors and nurses and enrich the sense of pharmacist’s accomplishment. Many previous studies have mentioned the need for pharmacists to strengthen communication with other medical occupations [6, 8–10, 30] to obtain more direct and timely patient information. Proper understanding can also illustrate that pharmacists consider and make drug recommendations from multiple perspectives, essential in providing high-quality care in interdisciplinary collaborations.

Finally, respondents offered several thoughts on pharmacist involvement in HBPC. They hope that the difficulties encountered by existing pharmacists in the field of home care services can be gradually improved with the assistance of government authorities to create a home-based field suitable for pharmacists to exert their expertise. Much literature also emphasises the importance of pharmacist education according to their area of expertise [9, 18]. In addition, some respondents are currently serving as cadres of the pharmacist association and hope that the association can plan and arrange relevant courses and training for pharmacists to enter the field of HBPC to solve the problem of insufficient staffing. Notably, Pallium Canada, a non-profit organisation, is dedicated to enhancing healthcare providers’ palliative care skills through its Learning Essential Approaches to Palliative Care programme [35, 36]. This initiative offers interprofessional courses addressing diverse care settings, covering essential topics like pain management and crucial conversations about palliative needs. Pallium’s approach, highlighting the interprofessional and multidisciplinary essence of palliative care, serves as a valuable model for other countries to learn from. Finally, establishing an improved environment for pharmacists supporting home care is crucial to ensure their continued excellent work, alignment with their initial aspirations, and dedication to the HBPC field.

### Study strengths and weaknesses/limitations

The study has some limitations. First, the small sample size may not fully represent the overall situation of the pharmacists participating in palliative home care in Taiwan. However, the volume of HBPC services in Taiwanese communities is still far less than that of hospital-based palliative care teams. Therefore, with the efforts of researchers, we explored and interviewed pharmacists who provide HBPC in southern Taiwan so the results should also be represented and valuable. Second, the respondents have some rapport with our research team, therefore, the pharmacists did not mention the negative aspects of working with clinical teams, which could introduce bias. Thus, a nationwide questionnaire survey is recommended to explore Taiwan’s overall community pharmacists’ perceptions of participation in HBPC.

## Conclusions

Community pharmacists play a crucial role in promoting and popularising HBPC. However, encouraging the input of community pharmacists and the preparation and supply of controlled drugs is essential for providing palliative care and is the first hurdle for community pharmacists to overcome. Once this threshold is crossed, many community pharmacists can fully play to their professional value and provide home palliative care, including medication consultation, health education, and polypharmacy control, which are also crucial for medication safety. Therefore, the health department should strive to improve the related difficulties, encourage more community pharmacists to invest in HBPC, and earnestly implement the spirit of community palliative care.

## Data Availability

The datasets used and/or analysed during the current study are available from the corresponding author on reasonable request.
